# Validation of a prognostic blood-based sphingolipid panel for men with localized prostate cancer followed on active surveillance

**DOI:** 10.1186/s40364-024-00678-7

**Published:** 2024-11-09

**Authors:** Justin R. Gregg, Lisa Newcomb, Ranran Wu, Jennifer Dennison, John W. Davis, Curtis Pettaway, Louis Pisters, John F. Ward, Brian F. Chapin, Lisly Chéry, Ahmet Urkmez, Andrew M. Fang, Noel Higgason, Patricia Troncoso, Carrie R. Daniel, Christopher Logothetis, Timothy C. Thompson, Andrew W. Hahn, Menghan Liu, Yingye Zheng, Dan W. Lin, Samir Hanash, Ehsan Irajizad, Johannes Fahrmann

**Affiliations:** 1 The University of Texas MD Anderson Cancer Center, 1515 Holcombe Blvd, Houston, TX 77030 US; 2 University of Washington and Fred Hutchinson Cancer Center, 1100 Fairview Ave N, Seattle, WA 98109 US

**Keywords:** Active surveillance, sphingolipids, biomarker, prostate cancer

## Abstract

**Background:**

We previously reported that increases in circulating sphingolipids are associated with elevated risk of biopsy Gleason grade group (GG) upgrading in men on Active Surveillance (AS) for prostate cancer. Here, we aimed to validate these findings and establish a blood-based sphingolipid biomarker panel for identifying men on AS who are at high-risk of biopsy GG upgrading.

**Methods:**

Men diagnosed with low- or intermediate-risk prostate cancer in one of two AS cohorts (CANARY PASS and MDACC) were followed for GG upgrading after diagnostic and confirmatory biopsy. The PASS cohort consisted of 544 patients whereas the MDACC Cohort consisted of 697 patients. The number of patients with GG upgrading during course of study follow-up in the PASS and MDACC cohorts were 98 (17.7%) and 133 (19.1%), respectively. Plasmas collected prior to confirmatory biopsy were used for mass spectrometry-based quantitation of 87 unique sphingolipid species. A neural network layer based on 21 sphingolipids was developed in the CANARY PASS cohort for predicting biopsy GG upgrading. Tertile-based thresholds for low-, intermediate-, and high-risk strata were subsequently developed for the sphingolipid panel as well as a model that combined the sphingolipid panel with PSA density and rate of core positivity on diagnostic biopsy. The resultant models and risk thresholds for GG upgrading were validated in the MDACC cohort. Performance was assessed using Cox proportional hazard models, C-index, AUC, and cumulative incidence curves.

**Results:**

The sphingolipid panel had a HR (per unit standard deviation increase) of 1.36 (95% CI: 1.07–1.70) and 1.35 (95% CI: 1.11–1.64) for predicting GG biopsy upgrading in the PASS and MDACC cohort, respectively. The model that combined the sphingolipid panel with PSA density and rate of core positivity achieved a HR of 1.63 (95% CI: 1.33-2.00) and 1.44 (1.25–1.66), respectively. Tertile-based thresholds, established in the PASS cohort, were applied to the independent MDACC cohort. Compared to the low-risk group, MDACC patients in the high-risk strata had a GG biopsy upgrade HR of 3.65 (95% CI: 2.21–6.02), capturing 50% of the patients that had biopsy upgrading during study follow-up.

**Conclusions:**

The sphingolipid panel is independently associated with GG biopsy upgrading among men in two independent AS cohorts who have previously undergone diagnostic and confirmatory biopsy. The sphingolipid panel, together with clinical factors, provides a potential means for risk stratification to better guide clinical management of men on AS.

**Supplementary Information:**

The online version contains supplementary material available at 10.1186/s40364-024-00678-7.

## Background

Active surveillance (AS) is the preferred management option for men diagnosed with low risk prostate cancer, and is an option for select men with intermediate risk disease [1]. While clinically safe, a very small proportion of men are at risk of future metastases on AS [2, 3]. Concern regarding disease progression and future metastasis form the basis of current AS protocols, all of which rely upon invasive biopsies as a gold standard in monitoring disease [4]. Gleason grade group (GG) biopsy upgrading remains the most common reason for delayed radical therapy on AS, which places men at risk of treatment-related changes to quality of life and affects an estimated 30–40% of men initiated on surveillance [5]. 

Clinical factors [6, 7] and risk calculators [8] have been developed to aid in determining men at increased risk for biopsy upgrading and, conversely, those who may be candidates for de-escalation of surveillance intensity [9]. However, they lack discriminatory power and have not resulted in guideline-based de-escalation strategies. Further, studies that evaluate the use of blood based [10, 11] and tissue based biomarkers [12] to augment clinical factors in predicting biopsy upgrading on AS have yielded only incremental improvements.

Previously, we demonstrated that increases in a circulating sphingolipids, a discrete sub-class of lipids, are associated with increased risk of GG biopsy upgrade among men on AS [13]. Studies by others have also reported circulating sphingolipids to be associated with poor clinical outcomes in patients with localized and metastatic prostate cancer [14]. Mechanistically, we demonstrated that Caveolin-1 (Cav-1), a protein that functions in organizing cell membrane microdomain composition and signal transduction, rewires prostate cancer cell metabolism towards increased uptake of circulating sphingomyelins (a type of sphingolipid). This process supports increased synthesis of glycosphingolipids that are subsequently secreted in particles that contain both Cav-1 and mitochondrial components, preventing the buildup of damaged mitochondria and subsequent cancer cell death [13]. 

In the current study, we aimed to confirm the association between plasma levels of sphingolipids and GG biopsy upgrade in the Prostate Active Surveillance Study (PASS). We subsequently developed a sphingolipid panel together with clinical risk markers for risk stratification of GG upgrading. Validation of the models was performed using an independent set of prospectively collected plasmas from patients enrolled in the MD Anderson Cancer Center active surveillance program [13].

## Methods

### Specimen sets

The Canary Prostate Active Surveillance Study (PASS) is a prospective (ClinicalTrials.gov NCT00756665) study enrolling men diagnosed with localized prostate cancer who have opted for AS from 2008 to 2019. All men with localized disease who chose active surveillance for management were eligible [15]. In the PASS cohort, PSA was measured every 3 months, clinic visits occurred every 6 months, and ultrasound guided biopsy (at least 10 cores) was performed 6–12 months and 24 months after diagnosis, then every 2 years. All biopsy slides were read by genitourinary pathologists at study sites [15].

The MD Anderson Cancer Center (MDACC) active surveillance cohort included men who were enrolled on a prospective AS protocol (NCT00490763) from 2006 to 2014 at single institution and who had over 6 months of follow-up (following confirmatory biopsy). Men with localized disease and GG1-3 disease who chose active surveillance were eligible [7]. Men were followed with biannual digital rectal exam, laboratory testing (serum PSA, testosterone), and 11 core systematic biopsies every 1–2 years [7]. All biopsy slides were read by genitourinary pathologists at MDACC. In both cohorts, MRI performance (and fusion biopsy) was not included as part of the protocol, though MRI was permitted if desired by the treating physician. Patients in both cohorts were followed for biopsy GG upgrading, defined as any increase in biopsy-based pathologic GG when compared to previously completed baseline or confirmatory biopsy.

Plasma specimens used for metabolomics assays were collected from patients enrolled in the respective cohorts during study follow-up, typically before (within 6 months) or at time of the first on-study (confirmatory) biopsy.

### Lipidomic analyses

Assaying of plasma sphingolipids, including sphingomyelin, ceramides, associated glycosphingolipid, and sulfatides, was conducted using a Waters Acquity™ UPLC system coupled to a Xevo G2-XS quadrupole time-of-flight (qTOF) mass spectrometer as previously described [13]. Chromatographic separation was performed using a C18 (Acquity™ UPLC HSS T3, 100 Å, 1.8 μm, 2.1 × 100 mm, Water Corporation, Milford, U.S.A) column at 55 °C. The mobile phases were (A) water, (B) Acetonitrile, (C) 2-propanol and (D) 500mM ammonium formate, pH 3. A starting elution gradient of 20% A, 30% B, 49% C and 1% D was increased linearly to 10% B, 89% C and 1% D for 5.5 min, followed by isocratic elution at 10% B, 89%C and 1%D for 1.5 min and column equilibration with initial conditions for 1 min.

Mass spectrometry data were acquired using ‘sensitivity’ mode in positive and negative electrospray ionization mode within 100–2000 Da. For the electrospray acquisition, the capillary voltage was set at 1.5 kV (positive), 3.0 kV (negative), sample cone voltage 30 V, source temperature at 120° C, cone gas flow 50 L/h and desolvation gas flow rate of 800 L/h with scan time of 0.5 s in continuum mode. Leucine Enkephalin; 556.2771 Da (positive) and 554.2615 Da (negative) was used for lockspray correction and scans were performed at 0.5 min. The injection volume for each sample was 3µL. The acquisition was carried out with instrument auto gain control to optimize instrument sensitivity over the samples acquisition time.

### Data processing

LC-MS and LC-MSe data were processed using Progenesis QI (Nonlinear, Waters). Peak picking and retention time alignment of LC-MS and MSe data were performed using Progenesis QI software (Nonlinear, Waters). Data processing and peak annotations were performed using an in-house automated pipeline as previously described [13, 16–18]. Annotations were determined by matching accurate mass and retention times using updated customized libraries created from authentic standards and by matching experimental tandem mass spectrometry data against the NIST MSMS, LipidBlast or HMDB v3 theoretical fragmentations. To correct for injection order drift, each feature was normalized using data from repeat injections of quality control samples collected every 10 injections throughout the run sequence. Measurement data were smoothed by Locally Weighted Scatterplot Smoothing (LOESS) signal correction (QC-RLSC) as previously described [13, 16–18]. 

### Statistical analyses

Univariable Cox Proportional hazard models were initially used to evaluate associations between individual sphingolipids with disease progression (defined here as GG biopsy upgrade following confirmatory biopsy). The event time was defined as the time from date of specimen collection to date of GG biopsy upgrade. Patients were censored at prostate cancer treatment (with or without GG upgrade), voluntary withdrawal, or loss to follow-up following biopsy. To test for the proportionality of hazard assumption of a Cox regression, we used the method of Patricia and Grambsch in all models [19]. 

To establish a sphingolipid panel for GG biopsy upgrading, we leveraged lipidomic profiles generated using plasma samples from the PASS cohort (Supplementary Figure S1). Eight different models, including deep learning model (DLM; from h2o package [20]), Conditional Non-Parametric Survival Estimator (from akritas package in R [21]), Cox-Time Survival Neural Network (from coxtime package in R [22]), Cox melding with top 20 features, Survival Neural Network (from DeepHit package in R [23]), Deep Survival Neural Network (from Deepsurv package in R [24]), Logistic-Hazard Survival Neural Network [25], and PC-Hazard Survival Neural Network [26] were used to identify patients on AS at increased risk of GG biopsy upgrade. Individual model performance was evaluated by Hazard Ratio, C-index, and AUC.

A deep learning model (DLM) with 3 hidden layers and 6 nodes in each layer was selected for modeling the 21-marker ‘sphingolipid panel’ based on AUC and Hazard Ratio. Additional details regarding methodology are provided in Supplementary Table S2. Univariable and Multivariable Cox Proportional hazard models were used to assess performance of the derived sphingolipid panel and GG biopsy upgrade as a singular variable and when adjusting for patient and tumor characteristics. Variables that retained statistical significance in multivariable modeling (sphingolipid panel, PSA density, and percentage (%) positive cores), were subsequently used for developing a combined model using Cox regression. Multiple hypothesis testing adjusted p values from likelihood ratio testing for each feature were evaluated to investigate the importance of the features in the combined model.

We further dichotomized men on AS into high-, intermediate-, or low-risk strata based on combined model score tertiles. Models and corresponding cut points were locked and subsequently validated using specimens from an independent cohort of men on AS from MDACC (MDACC Set #1). Performance was further evaluated for external testing using a prior set of metabolomic data generated from men on AS [13] as well as in the combined MDACC cohort (Set #1 and #2) (Supplementary Figure S1). We note that not all patients had complete clinical information, precluding combined model evaluation on the entire specimen set. Cumulative incidence curves based on the different risk strata were generated using the ‘Survminer’ package in R statistical software (https://www.r-project.org/). Reported p-value for cumulative incidence curves estimated from the chi-square test with one-degree of freedom with the corresponding null hypothesis that there is no linear trend between the order of the groups and the median survival time. Model performance was further assessed in a subset of MDACC patients with available MRI results following stratification by MRI risk and lesion number.

Power calculations and cohort data stratified by progression status for the PASS Cohort, MDACC Set #1 and MDACC Set #2 are provided in Supplementary Tables S12 and S13, respectively.

## Results

### Application of artificial intelligence to circulating sphingolipid profiles to establish an improved risk prediction model in the PASS Cohort

Our prior work demonstrated that elevated plasma sphingolipids were associated with prostate cancer progression [13]. We assembled a cohort of plasma samples collected from 547 men on AS from the Canary PASS study to further evaluate the association between circulating sphingolipid species, including those described in our prior work [13] as well as additional sphingolipids not previously considered, and biopsy upgrade among men on AS. Table 1 lists baseline demographic, oncologic and follow-up data. Of the 544 patients, median age was 67 years (interquartile range (IQR): 58–70) and 467/544 (85.8%) had Gleason GG 1 disease, at most, on diagnostic or on-study confirmatory biopsy. Median follow-up in the Canary PASS study was 2.1 years (IQR: 1.4–4.5 years), and 98 (18.0%) of the 544 patients had a biopsy GG upgrading during study follow-up.

**Table 1:** Patient characteristics of the Canary PASS cohort and MDACC Validation Cohort

Variable	Canary PASS	MDACC Set #1	MDACC Set #2	*P*-value comparing Canary PASS and MDACC Set #1**
**N**	544	238	459	459
**Age**,** yrs (median**,** IQR)**	67 (58–70)	63 (58–68)	63 (58–69	0.18
**PSA Density (median**,** IQR)**	0.086 (0.069–0.15)	0.10 (0.06–0.15)	0.09 (0.06–0.14)	0.98
**Highest Gleason Grade Group**,** diagnostic or confirmatory biopsy (N**,** %)**				
** 1**	467 (85.8)	204 (90.0)	401 (87.4)	0.02
** 2**	74 (13.6)	27 (11.3)	53 (11.5)	
** 3**	3 (< 1)	7 (2.9)	5 (1.1)	
**Percentage of positive cores at diagnosis (median**,** IQR)**	10.0 (8.3–16.7)	9.1 (8.3–16.7)	9.1 (8.3–16.7)	0.18
**BMI (median**,** IQR)**	26.9 (24.7–31.0)	29.1 (26.3–32.6)	28.6 (26.1–31.6)	< 0.01
**Race**				
** White**	477 (87.7)	198 (83.2)	376 (81.9)	0.049
** Black**	38 (7.0)	15 (6.3)	36 (7.8)	
** Other/Unknown**	29 (5.3)	25 (10.5)	47 (10.2)	
**Statin (N**,** %)**				
** Yes**	238 (43.8)	117 (49.2)	217 (47.3)	0.18
** No**	306 (56.3)	121 (50.8)	242 (52.7)	
**Diabetes (N**,%)*				
** Yes**	48 (8.8%)			
** No**	496 (91.2%)			
**Follow-up**,** yrs (median**,** IQR)**	2.1 (1.4–4.5)	3.5 (1.0-5.1)	3.0 (1.0-4.5)	< 0.01
**Total biopsy number**,** excluding diagnostic (median**,** IQR)**	2 (1–2)	2 (1–3)	2 (2–3)	< 0.01
**Gleason grade group upgrade (any) during follow-up (N**,** %)**				
** Yes**	98 (18.0)	33 (13.9)	98 (21.4)	0.10
** No**	446 (82)	215 (90.3)	361 (78.6)	
**Gleason grade group upgrade (GG3 or higher) during follow-up (N**,** %)**				
** Yes**	39 (7.2)	7 (2.9)	35 (7.6)	0.02
** No**	505 (92.8)	231 (97.1)	424 (92.4)	

*Baseline diabetes status not available for MDACC cohorts

**These cohorts compared as MDACC Set #1 was first evaluated using cut points established in Canary PASS as it is independent from previously reported patient data (MDACC Set #2; Vykoukal et al. Nat Commun 2020). Categorical variables were compared using Fisher’s Exact Test for two-class comparisons and χ2 tests for trends if more than two class comparison. Continuous variables were compared using Student T-test or Wilcoxon Rank Sum Tests depending on data normality

Assaying of sphingolipids was performed using ultra high-pressure liquid chromatography mass spectrometry (UHPLC-MS). A total of 87 uniquely annotated sphingolipids were quantified. Increases in individual sphingolipid species tended to be associated with GG biopsy upgrade on AS (Supplemental Dataset S1) [13]. Next, we evaluated eight different machine learning algorithms to establish a panel of sphingolipids for predicting GG upgrading. A neural network with 3 layers and 32 nodes in each layer based on 21 sphingolipids (hereon referred to as the “sphingolipid panel”) achieved the highest performance in the PASS cohort, yielding a hazard ratio (HR) of 1.36 (95% CI: 1.07–1.70) per unit standard deviation (SD) increase based on univariable Cox proportional hazards analysis (Supplementary Tables S1-2). Multivariable Cox proportional hazards models that accounted for relevant clinical (e.g. age, BMI, statin use [see Supplementary Figure S2 for additional BMI and statin data]) and oncologic (e.g. biopsy characteristics) factors demonstrated that the sphingolipid panel was independently associated with biopsy GG upgrade, with an HR of 1.33 (95% CI: 1.05–1.70) per SD increase (Supplementary Table S3). Cox proportional hazard assumptions were met. Of note, sphingolipid panel scores did not exhibit statistically significant associations with BMI (Pearson r: -0.07). Sphingolipid panel scores tended to be lower in AS patients on statins (Supplementary Figure S2). Based on multivariable Cox proportional models, PSA density (PSAD) (HR 1.36, 95% CI 1.16–1.61) and diagnostic biopsy core positivity rate (HR 1.27, 95% CI 1.00-1.61) were also found to be independently associated with biopsy GG upgrading (Supplementary Table S3).

To better inform clinical decision making, we assessed the contributions of the three independent factors most strongly associated with biopsy progression - the sphingolipid panel, PSAD and % positive core biopsy rate (Supplementary Figure S3; Supplementary Table S3-4) - for identifying men on AS at increased risk of GG biopsy upgrade. A model that combined these three factors had an HR 1.63 (95% CI: 1.33-2.00) per unit SD increase for GG upgrade (Supplementary Table S5; Supplementary Figure S4).

**Fig. 1:**
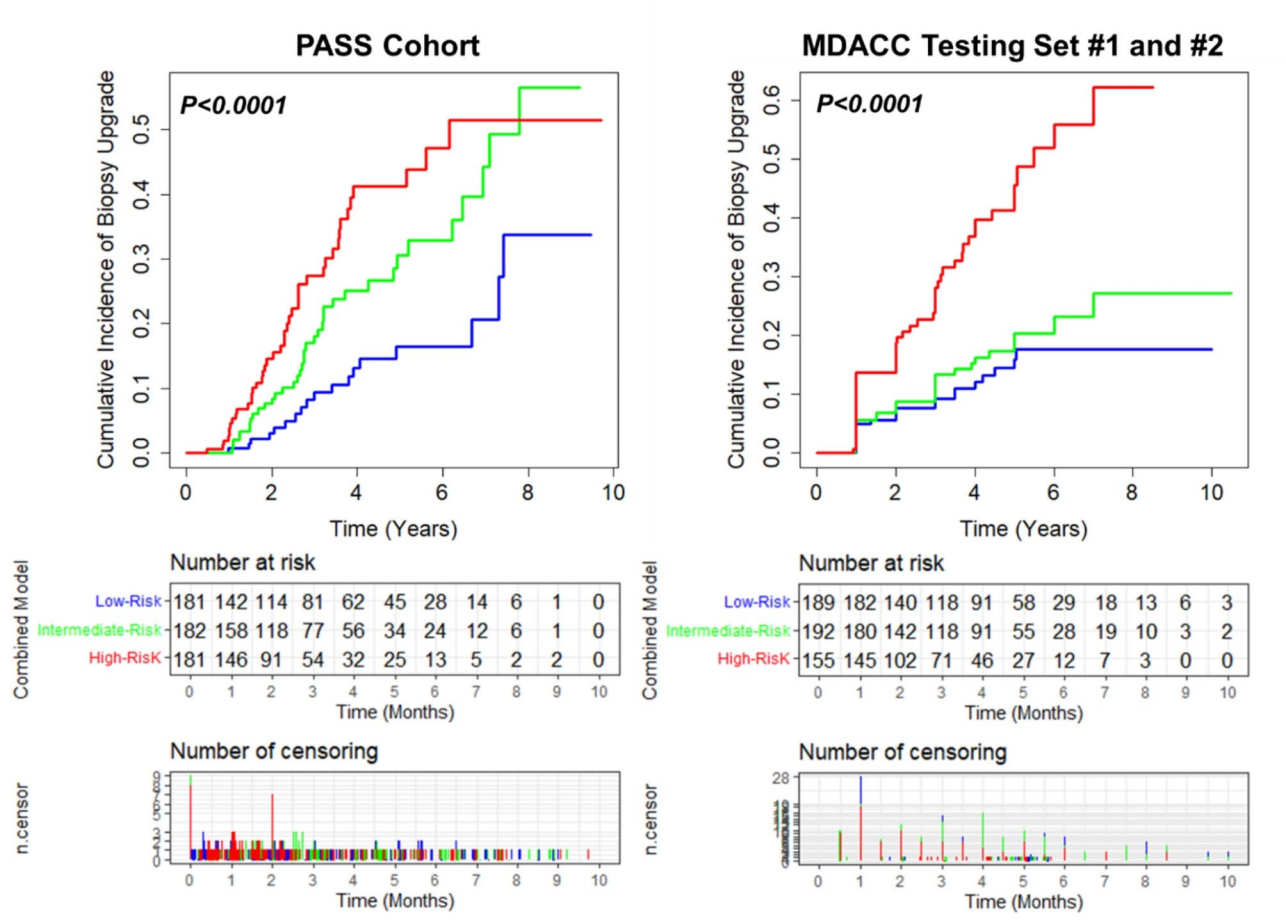
Cumulative incidence curves for GG biopsy upgrade based on combined model at high-, intermediate-, and low-risk strata in the PASS and MDACC Cohorts. Risk tables, including censoring events, are provided beneath. Censoring was attributed to GG upgrading, prostate cancer treatment, voluntary withdrawal from AS, or loss to follow-up following biopsy

We next placed men on AS into three risk strata, high-, intermediate-, or low-risk, based on combined model (sphingolipid panel + PSAD + positive core biopsy rate) score tertiles (Supplementary Table S6). Compared to the low-risk group, patients in the high- or intermediate-risk strata had significantly higher risk of biopsy GG upgrade (HR 3.17 (95% CI: 1.84–5.46) and HR 2.05 (95% CI: 1.18–3.58), respectively), and this association was more pronounced than individual factors (Table 2). Cumulative incidence curves further demonstrated that patients in the high-risk strata had the highest incidence of biopsy upgrade (23.2%), compared to those in the intermediate- or low-risk strata (Table 2; Fig. 1), and accounted for 42 out of the 98 patients who had GG upgrading during study follow-up.

**Table 2:** Performance estimates of the sphingolipid panel, PSA density, % positive core biopsy and the model that combines all three at different risk strata for predicting biopsy upgrade for men on AS in the Canary PASS and the combined MDACC Cohort (Testing Set #1 and #2)

		Canary PASS						
Variable	Strata	# of Events	# of Patients	Biopsy Upgrade, *N* (%)	No Biopsy Upgrade, *N* (%)	Median time to Upgrade, Months (IQR)	*P*- value†	Hazard Ratio (95% CI)
**Combined**	**High-Risk**	**98**	**544**	**42 (23.2)**	**139 (76.8)**	**27.0 (16.5–39.6)**	**0.0017**	**3.17 (1.84–5.46)**
	**Intermediate-Risk**			**37 (20.3)**	**145 (79.7)**	**33.1 (19.4–47.9)**		**2.05 (1.18–3.58)**
	**Low-Risk**			**19 (10.5)**	**162 (89.5)**	**35.9 (24.6–58.9)**		**reference**
Sphingolipid Panel	High-Risk	98	544	45 (24.9)	136 (75.1)	28.9 (17.5–42.8)	0.0017	1.84 (1.11–3.07)
	Intermediate-Risk			31 (17)	151 (83)	28.3 (18.4–36.8)		1.28 (0.74–2.22)
	Low-Risk			22 (12.2)	159 (87.8)	35.9 (24.6–46.8)		reference
PSA Density	High-Risk	98	544	38 (21.0)	143 (79.0)	24.5 (17.9–41.6)	0.218	1.91 (1.18–3.10)
	Intermediate-Risk			31 (17.0)	151 (83.0)	31.2 (18.6–42.7)		1.15 (0.69–1.91)
	Low-Risk			29 (16.0)	152 (84.0)	30.6 (18.1–41.0)		reference
% Core Biopsy	High-Risk	98	544	34 (18.8)	147 (81.2)	30.4 (17.5–41.6)	0.494	2.27 (1.37–3.74)
	Intermediate-Risk			35 (19.2)	147 (80.8)	26.5 (17.9–38.4)		1.33 (0.81–2.19)
	Low-Risk			29 (16.0)	152 (84.0)	33.7 (24.7–68.6)		reference

		**MDACC AS (Set #1 and Set #2 [Vykoukal et al. 2020])**						
**Variable**	**Strata**	**# of** **Events**	**# of** **Patients**	**Biopsy Upgrade**,** N (%)**	**No Biopsy Upgrade**,** N (%)**	**Median time to Upgrade**, **Months (IQR)**	**P-** **value†**	**Hazard Ratio** **(95% CI)**
**Combined**	**High-Risk**	**102**	**536**	**51 (32.9)**	**104 (67.1)**	**24.0 (12.0–42.0)**	**< 0.0001**	**3.65 (2.21–6.02)**
	**Intermediate-Risk**			**29 (15.1)**	**163 (84.9)**	**24.0 (12.0-44.5)**		**1.33 (0.77–2.32)**
	**Low-Risk**			**22 (11.6)**	**167 (88.4)**	**24.0 (12.0-43.5)**		**reference**
Sphingolipid Panel	High-Risk	131	707	55 (22.7)	187 (77.3)	24.0 (12.0–42.0)	0.014	1.78 (1.17–2.72)
	Intermediate-Risk			41 (18.9)	176 (81.1)	24.0 (12.0-37.1)		1.31 (0.83–2.05)
	Low-Risk			35 (14.1)	213 (85.9)	24.0 (12.0–36.0)		reference
PSA Density	High-Risk	127	642	54 (27.3)	144 (72.7)	24.0 (12.0-36.2)	0.001	2.31 (1.51–3.53)
	Intermediate-Risk			37 (18.3)	165 (81.7)	24.0 (12.0–42.0)		1.35 (0.85–2.13)
	Low-Risk			36 (14.9)	206 (85.1)	24.0 (12.0-46.5)		reference
% Core Biopsy	High-Risk	102	538	26 (31)	58 (69)	25.0 (12.0-44.6)	0.001	2.56 (1.55–4.23)
	Intermediate-Risk			39 (19.8)	158 (80.2)	24.3 (12.0–42.0)		1.49 (0.95–2.34)
	Low-Risk			37 (14.4)	220 (85.6)	16.5 (12.0–42.0)		reference

† χ2 test for trend 2-sided p-value

### Performance of the sphingolipid panel, the combined model, and corresponding risk thresholds for biopsy upgrade in the MDACC testing sets

Validation of the sphingolipid panel as well as the combined model (sphingolipid panel + PSAD + positive core biopsy rate), using fixed model coefficients developed in the PASS cohort, was performed in an independent set of plasmas prospectively collected from 238 patients on AS at MDACC (MDACC Set #1; Table 1). Median age was 63 years (IQR 58–68) and 204/238 (90.0%) had GG 1 disease, at most, on diagnostic or on-study confirmatory biopsy. Median follow-up of the cohort was 3.5 years (IQR 1.0-5.1 years), and 33 (13.9%) patients had a biopsy GG upgrading during study follow-up.

The sphingolipid panel was associated with risk of biopsy GG upgrade (HR 1.80, 95% CI: 1.18–2.74 per unit SD increase; Supplementary Table S7). The combined model that included the sphingolipid panel, PSAD and positive core biopsy rate yielded an HR of 3.07 (95% CI: 2.07–4.54) for biopsy GG upgrade (Supplementary Table S7-8; Supplementary Figure S4-5).

To increase sample size, we further combined MDACC Set #1 with MDACC Set #2, which included 459 patient samples used in the initial discovery of the association between sphingolipids and biopsy GG upgrade on AS (Table 1; Fig. 1; Supplementary Tables S9-10; Supplementary Figure S6) [1]. In the combined MDACC cohort (Set #1 and #2), the sphingolipid panel had a HR (per unit SD increase) of 1.35 (95% CI: 1.01–1.58) whereas the combined panel had a HR of 1.44 (95% CI: 1.25–1.66) (Supplementary Table S5).

We next stratified patients into high-, intermediate-, or low-risk groups using fixed cut points derived in the PASS cohort (Supplementary Table S6). Compared to the low-risk group, patients in the high-risk strata had an HR of 3.65 (95% CI: 2.21–6.02) (Table 2). Cumulative incidence curves further demonstrated that patients in the high-risk strata had the highest incidence of biopsy upgrade (32.9%), compared to those in the intermediate- or low-risk strata (Table 2; Fig. 1), and accounted for 50% of all patients (51 out of 102) who had GG upgrading during study follow-up.

We additionally evaluated the performance of combined model for risk stratification of biopsy GG upgrading for the subset of AS patients who underwent MRI. In the MDACC AS cohort, individuals with MRI PIRADS 3 + tended to have higher risk of biopsy GG upgrade compared to patients with PIRADS 0–2 (HR: 1.43 [0.59–3.46]; 2-sided p-value: 0.42) (Supplementary Table S11, Supplementary Figure S7). In contrast, high-risk patients based on combined panel scores > 3.2116 (Supplementary Table S6) had an HR of 12.13 (95% CI: 3.08–47.78) in the context of a negative MRI (Supplementary Table S11).

## Discussion

We describe a plasma-based, sphingolipid panel that is associated with risk of GG biopsy upgrading, the most common reason for delayed treatment on AS [6], in multiple mature AS cohorts. When combined with clinical factors that are independently associated with GG upgrading, the panel effectively risk-stratified men into groups at increased (and decreased) risk of biopsy upgrading. These findings build on our prior work demonstrating alterations in sphingolipid metabolism that are present in both men with PCa and PCa model systems, [13] and offer further evidence that the sphingolipid panel described here may represent a useful biological correlate of localized PCa progression risk with improved predictive capacity.

In our prior studies, we reported that increases in circulating sphingolipids were associated with GG upgrading in men on active surveillance. Biologically, we attributed increases in circulating sphingolipids to an underlying onco-metabolic pathway that is active in prostate cancer cells and that is regulated by Cav1. Specifically, we found coordinated activities through which Cav1 rewires prostate cancer cells towards exogenous scavenging of sphingomyelin, increased cancer cell catabolism of sphingomyelin to ceramide, and subsequent glycosylation to glycosphingolipid derivatives [13]. We further showed that increased Cav1-mediated glycosphingolipid synthesis supports biogenesis and release of (glyco)sphingolipid-enriched extracellular vesicles (EVs) that enables removal of mitochondrial components to maintain mitochondrial quality control [13]. Targeting of this onco-metabolic pathway via repurposing of eliglustat, a selective small molecule inhibitor of glucosylceramide synthase (UGCG), elicited anti-cancer effects in vitro and attenuated tumor development in an orthotopic syngeneic RM-9 mouse model of prostate cancer. The anti-cancer effects of eliglustat were attributed to hyperaccumulation of ceramides in prostate cancer cells, resulting in induction of compensatory mitophagy and subsequent cancer cell death [13]. Thus, the sphingolipid panel reported herein may serve as a surrogate for the above described onco-metabolic pathway, which may be targetable through repurposing of available small molecule inhibitors of UGCG. We also note that this pathway is likely important among other malignancies, as we and others have reported increases in circulating sphingolipids in other cancers, such as breast cancer [27]. Our group is also investigating the potential for behavioral interventions, such as dietary change, as means to mitigate disease progression risk associated with an elevated sphingolipid signature, and this work is ongoing.

AS is increasingly utilized, and, while safe, places a myriad of burdens on patients and physicians, including frequent outpatient visits, [29] potential biopsy side effects, [30] costs of care, [31] and changes to quality of life associated with delayed radical treatment [32]. While such costs may be justifiable within the context of improved oncologic outcomes, a major challenge in approaching men with low and intermediate risk prostate cancer from an oncologic perspective is the generally indolent nature of the disease. Recent 15-year data from the randomized PROTECT trial demonstrated a rate of prostate cancer-specific death of 3.1% among men managed with AS, [3] which was not significantly different (both clinically and statistically) than the 2.2% and 2.9% rates of death seen in the surgical and radiotherapy arms, respectively. Thus, the performance demands of any risk stratification system (based on clinical factors, biomarkers, or a combination) that is designed to delineate AS candidates who would gain an oncologic survival benefit from early radical treatment are exceedingly high. Therefore, the identification of stratification systems, such as that described in this work, that may safely guide surveillance de-escalation among men on AS, while identifying individuals at increased risk, are sorely needed. This may include stratification of patients for more (or less) frequent PSA, MRI, and biopsy testing.

Multiple prior investigations have sought to elucidate associations between prostate cancer biomarkers and progression on AS, though few have demonstrated the ability to stratify men at risk of GG biopsy upgrading. In terms of plasma-based markers, the 4K score is associated with risk of upgrading at time of initial repeat biopsy, though is not associated with biopsy upgrading over time [11]. Similarly, Prostate Health Index is associated with initial biopsy upgrading, but not subsequent biopsy results [10]. When considering tissue-based markers (which require invasive biopsy to complete), a study completed in the PRIAS trial demonstrated that tumor PTEN status is associated with GG upgrading and adverse pathology at prostatectomy, though the study was limited in size and has not been externally validated [28]. To contrast, a study in the Canary PASS cohort showed that a 17-gene Genomic Prostate Score was not independently associated with biopsy upgrading [12] and a study from the MUSIC collaborative evaluating the Decipher Prostate Biopsy Assay did not demonstrate an association with GG upgrading (though high scores were associated with shorter time to treatment and treatment failure) [29]. While these (and other) tissue-based markers are therefore associated with oncologic outcomes such as post-prostatectomy pathology and metastasis, their predictive value within the context of newly diagnosed prostate cancer managed on AS remains unclear [30]. To date, PSAD remains the marker that is most consistently associated with biopsy upgrading on AS [31–33] and it is included in guideline-based risk stratification of men who are diagnosed with GG 1 disease [1]. Importantly, PSAD was associated with GG upgrading in our cohort; however, the combination of PSAD, core biopsy positivity, and the sphingolipid panel demonstrated improved risk stratification (Table 2) which may be leveraged through the use of marker-based cut points. Future studies using the cut points described in this work to select groups for less frequent visits, tests, and biopsies, while continuing to monitor those at increased risk of GG biopsy progression, may improve the efficiency of low risk prostate cancer management, while maintaining the safety of deescalated care algorithms.

Ultimately, future studies evaluating GG progression risk and biopsy frequency among men on AS need to incorporate the remaining predictor that shows promise in discriminating potentially aggressive from indolent disease among those with localized prostate cancer: magnetic resonance imaging (MRI). Prostate MRI with guided biopsy is established as a more effective means to diagnosis GG 2 or higher prostate cancer when compared to systematic biopsy [34] and is increasingly used among men on AS [35, 36]. Further, a randomized study demonstrated that rates of upgrading on AS decreased when MRI-guided biopsy was used [37]. While the current work offers the benefits of: (1) AS performance in a large number of patients, (2) extensive follow-up (having been initiated in the mid-2000s) [15, 38] *after* confirmatory biopsy performance, and, (3) consistent biopsy techniques, a limitation of this study is that most patients did not undergo MRI-based stratification or fusion biopsy. Notably, work in the Canary PASS AS cohort demonstrated that prostate MRI findings did not improve upon clinical factors when assessing biopsy-based GG upgrading risk within 12 months of MRI performance [39]. Similarly, sphingolipid panel performance remained consistent among a subgroup of MDACC patients stratified by MRI results in the current study. These data suggest that the sphingolipid panel, alone and in combination with factors such as PSAD, is likely independently associated with GG upgrade risk. However, future studies that incorporate MRI and targeted biopsy results along with sphingolipid panel levels and other clinical factors are needed to determine the optimal patients in whom de-escalation may occur, especially considering that MRI estimates of tumor characteristics and size may prove to be stronger predictors of upgrading risk than clinical variables such as prostate core positivity that are included in the current work. In terms of other limitations, our current work lacks racial diversity, including in AA men (who are at increased risk of adverse prostate cancer outcomes) [2], potentially limiting the overall generalizability of our results. Our work is also limited by follow-up time, which is attributed to progression events, patients electing treatment, and patient preference to follow closer to home (as opposed to continued visits at tertiary care centers). However, these limitations are present among most mature active surveillance cohorts from this time and did not preclude robust analyses performed in this work. We also acknowledge that factors such as obesity and statin use and metabolic disorders (e.g. diabetes) alter lipid profiles, which may affect sphingolipid levels [40]. Nonetheless, we emphasize that multivariable models taking these factors into account revealed an independent association between the sphingolipid panel and GG upgrading.

## Conclusions

In conclusion, the sphingolipid panel may serve as a useful adjunct in determining patient management among men with low- and intermediate-risk prostate cancer managed on AS. Future validation studies are needed to inform its potential use in management of men with prostate cancer managed on AS as a means to tailor follow-up and biopsy frequency among those at the highest (and lowest) risk of biopsy upgrading.

## Electronic Supplementary Material

Below is the link to the electronic supplementary material.

Supplementary Material 1

Supplementary Material 2

## Data Availability

Due to potential compromise of individual privacy a complete dataset is not available. However, data will be provided, in addition to statistical code, following individual requests to study authors.
