# Supplementary material for: Validation of a prognostic blood-based sphingolipid panel for men with localized prostate cancer followed on active surveillance
Source: Biomark Res. 2024 Nov 9;12:134. doi: 10.1186/s40364-024-00678-7 (PMC11550521; doi:10.1186/s40364-024-00678-7)
Supplement: Supplementary file 2 — Supplementary Material 2 [file 40364_2024_678_MOESM2_ESM.docx]

**Supplementary Datasets, Tables and Figures**

**Supplementary Dataset S1. Hazard ratios of quantified sphingolipid species for biopsy GG upgrade in the Canary Pass Cohort** (see separate document)

**Supplementary Table S1. Performance estimates of different deep-learning models evaluating association of sphingolipid panel with biopsy Gleason GG upgrade on AS.**

|  | **Performance** | | | |
| --- | --- | --- | --- | --- |
|  | **Training (PASS)** | | | |
| **Method** | **HR** | **P-value** | **C-index** | **AUC** |
| Neural Network (from H2O Package) | **1.30** | 0.014 | 0.521 | 0.608 |
| akritas (Conditional Non-Parametric Survival Estimator) | 0.66 | 0.881 | 0.465 | 0.471 |
| coxtime (Cox-Time Survival Neural Network) | 1.05 | 0.615 | 0.528 | 0.549 |
| Coxph (regular Cox with top 20 features) | 0.98 | 0.818 | 0.485 | 0.467 |
| DeepHit Survival Neural Network | 1.09 | 0.367 | 0.532 | 0.565 |
| Deepsurv Survival Neural Network | 0.94 | 0.582 | 0.471 | 0.452 |
| Logistic-Hazard Survival Neural Network | 0.99 | 0.957 | 0.525 | 0.536 |
| PC-Hazard Survival Neural Network | 1.03 | 0.747 | 0.519 | 0.509 |

Abbreviation: HR- hazard ratio per unit increase

**Supplementary Table S2. Deep learning model methods and sphingolipids selected for inclusion in the deep learning model.**

A DLM with 3 hidden layers and 6 nodes in each layer was selected for modeling the 21-marker metabolite panel based on AUC and Hazard Ratio. Grid-search was performed across hyperparameters to tune and find the best performing combination.[20] The method reported by Gedeon was used to assign importance score for variables included in the model. [20, 27, 28] This approach removes irrelevant or noisy signal by analyzing for the relative weight of each variable within the overall data matrix. An importance score is calculated by dividing the absolute value of the weight of an input connecting to an output by the total absolute value of all weights from that input. When applied in the deep learning model, this approach is recursively extended backwards through layers by taking the effect of a neuron on a connected node, then multiplying the derived weight by the effect of the given node on the target output and summing all connecting nodes.

$$P_{jk}= \frac{|w_{jk}|}{\sum_{r=1}^{nh} |w_{rk}|}$$

Here, $P_{jk}$represents the average contribution of a node j in a layer to a node k in the next layer. w is the weight on the connection and nh is the number of nodes in the next layer.

The contribution of an input neuron to an output is:

$$Q_{ik}= \sum_{r=1}^{nh} (P_{ir}\times P_{rk})$$

The below table shows sphingolipids selected for inclusion in the deep learning model:

| **Variable** | **Relative Importance** | **Scaled Importance** | **Relative Contribution** |
| --- | --- | --- | --- |
| Sphingomyelin(40:1) | 1.000 | 1.000 | 0.074 |
| Sphingomyelin(33:2) | 0.997 | 0.997 | 0.074 |
| Sphingomyelin(34:2) | 0.943 | 0.943 | 0.070 |
| C16 sulfatide | 0.905 | 0.905 | 0.067 |
| Sphingomyelin(39:1) | 0.783 | 0.783 | 0.058 |
| LactosylCeramide(18:1/16:0) | 0.780 | 0.780 | 0.058 |
| Sphingomyelin(42:2) | 0.697 | 0.697 | 0.052 |
| Sphingomyelin(33:1) | 0.672 | 0.672 | 0.050 |
| Sphingomyelin(40:3) | 0.632 | 0.632 | 0.047 |
| Sphingomyelin(41:1) | 0.615 | 0.615 | 0.046 |
| C22 sulfatide | 0.598 | 0.598 | 0.044 |
| Sphingomyelin(33:1) | 0.571 | 0.571 | 0.042 |
| Sphingomyelin(36:2) | 0.568 | 0.568 | 0.042 |
| Sphingomyelin(39:2) | 0.565 | 0.565 | 0.042 |
| Sphingomyelin(34:0) | 0.532 | 0.532 | 0.039 |
| LactosylCeramide(32:1) | 0.526 | 0.526 | 0.039 |
| C16(OH) Sulfatide | 0.459 | 0.459 | 0.034 |
| Sphingomyelin(32:1) | 0.458 | 0.458 | 0.034 |
| Sphingomyelin(40:2) | 0.401 | 0.401 | 0.030 |
| Sphingomyelin(32:2) | 0.394 | 0.394 | 0.029 |
| C22(OH) Sulfatide | 0.372 | 0.372 | 0.028 |

**Supplementary Table S3. Cox proportional hazard models evaluating association of clinicopathologic characteristics and the sphingolipid panel with biopsy Gleason GG upgrading in the Canary PASS cohort.**

|  | **Univariable¥** | | | **Multivariable** | | |
| --- | --- | --- | --- | --- | --- | --- |
| **Variable** | **Hazard Ratio** | **95% CI** | **2-sided P** | **Hazard Ratio** | **95% CI** | **2-sided P** |
| **Age** | 1.02 | 0.99-1.05 | 0.31 | 1.01 | 0.98-1.04 | 0.59 |
| **Ethnicity** |  |  |  |  |  |  |
| **African AmericanAmerican/Black** | Reference | | | Reference | | |
| **White** | 1.37 | 0.50-3.74 | 0.54 | 1.52 | 0.53-4.34 | 0.44 |
| **Other** | 2.21 | 0.62-7.83 | 0.22 | 2.38 | 0.64-8.89 | 0.20 |
| **BMI†** | 1.01 | 0.96-1.06 | 0.73 | 1.02 | 0.98-1.07 | 0.34 |
| **Diabetes** |  |  |  |  |  |  |
| **No** | Reference | | | Reference | | |
| **Yes** | 0.85 | 0.40-1.84 | 0.69 | 0.90 | 0.40-2.05 | 0.80 |
| **Statin Use** |  |  |  |  |  |  |
| **No** | Reference | | | Reference | | |
| **Yes** | 0.87 | 0.58-1.31 | 0.51 | 0.92 | 0.60-1.39 | 0.70 |
| **5ARI treatment** |  |  |  |  |  |  |
| **No** | Reference | | | Reference | | |
| **Yes** | 1.20 | 0.60-2.38 | 0.61 | 1.42 | 0.70-2.87 | 0.33 |
| **PSA Density†** | 1.34 | 1.14-1.57 | 0.0005 | 1.36 | 1.16-1.61 | 0.0002 |
| **GGG‡** | 1.47 | 0.84-2.55 | 0.18 | 1.06 | 0.56-2.02 | 0.85 |
| **Positive Core Biopsy %†** | 1.31 | 1.06-1.63 | 0.012 | 1.27 | 1.00-1.61 | 0.044 |
| **Sphingolipid Panel†** | 1.36 | 1.07-1.70 | 0.012 | 1.33 | 1.05-1.70 | 0.018 |

† per unit standard deviation increase; ‡ per unit increase

Global p (constant HR): 0.25

**¥** for univariable analyses, variables were analyzed as a single input into the Cox proportional hazard model

**Supplementary Table S4. Likelihood ratio tests in the Canary PASS cohort for the combined (sphingolipid panel + PSA density + percent positive core biopsies) model.**

| **Training Set** | **Likelihood ratio testing adjusted p-value** |
| --- | --- |
| Sphingolipid Panel | 0.021 |
| PSA Density | 0.002 |
| (%) Positive Core Biopsy | 0.030 |

**Supplemental Table S5. Univariable Cox proportional hazard models evaluating the association of the sphingolipid panel, PSA density, and percent positive core biopsies for Gleason GG upgrading in the Canary PASS, MDACC Set #1, MDACC Set #2 (Vykoukal et al. 2020), and the combined MDACC (Set #1 and Set #2) cohorts.** Each variable was analyzed separately.

| **Training Set (PASS)** | | | | |
| --- | --- | --- | --- | --- |
| **Variable** | **HR†** | **95% CI** | **2-sided P** | **C-Index (S.E.)** |
| Sphingolipid Panel | 1.36 | 1.07-1.70 | 0.012 | 0.599 (0.028) |
| PSA Density | 1.34 | 1.14-1.57 | 0.0005 | 0.621 (0.032) |
| (%) Positive Core Biopsy | 1.31 | 1.06-1.63 | 0.012 | 0.560 (0.033) |
| PSA Density + PCB | 1.49 | 1.23-1.81 | <0.0001 | 0.646 (0.027) |
| Sphingolipid Lipid Panel + PSA Density + PCB Model | 1.63 | 1.33-2.00 | <0.0001 | 0.674 (0.028) |
| **MDACC Set #1** | | | | |
| **Variable** | **HR†** | **95% CI** | **2-sided P** | **C-Index (S.E.)** |
| Sphingolipid Panel | 1.80 | 1.18-2.74 | 0.006 | 0.637 (0.053) |
| PSA Density | 1.74 | 1.29-2.35 | 0.0003 | 0.675 (0.054) |
| (%) Positive Core Biopsy | 1.47 | 1.12-1.94 | 0.006 | 0.567 (0.059) |
| PSA Density + PCB | 2.12 | 1.55-2.89 | <0.0001 | 0.705 (0.057) |
| Sphingolipid Lipid Panel + PSA Density + PCB | 3.07 | 2.07-4.54 | <0.0001 | 0.776 (0.048) |
| **MDACC Set #2 (Vykoukal et a. 2020)** | | | | |
| **Variable** | **HR†** | **95% CI** | **2-sided P** | **C-Index (S.E.)** |
| Sphingolipid Panel | 1.26 | 1.01-1.58 | 0.040 | 0.568 (0.033) |
| PSA Density | 1.14 | 1.00-1.30 | 0.06 | 0.610 (0.030) |
| (%) Positive Core Biopsy | 1.23 | 1.05-1.45 | 0.012 | 0.594 (0.037) |
| PSA Density + PCB | 1.20 | 1.02-1.42 | 0.031 | 0.612 (0.036) |
| Sphingolipid Lipid Panel + PSA Density + PCB | 1.28 | 1.07-1.54 | 0.009 | 0.629 (0.039) |
| **MDACC AS (MDACC Set #1 and #2 [Vykoukal et al. 2020])** | | | | |
| **Variable** | **HR†** | **95% CI** | **2-sided P** | **C-Index (S.E.)** |
| Sphingolipid Panel | 1.35 | 1.11-1.64 | 0.003 | 0.576 (0.028) |
| PSA Density | 1.19 | 1.06-1.32 | 0.002 | 0.615 (0.027) |
| (%) Positive Core Biopsy | 1.28 | 1.12-1.47 | 0.0003 | 0.582 (0.031) |
| PSA Density + PCB | 1.30 | 1.15-1.48 | <0.0001 | 0.616 (0.031) |
| Sphingolipid Lipid Panel + PSA Density + PCB | 1.44 | 1.25-1.66 | <0.0001 | 0.646 (0.032) |

Abbreviations: HR-hazard ratio; Cl- confidence interval; PCB- % positive core biopsy rate

† per unit standard deviation increase

**Table S6. Cut points for high-, intermediate-, and low-risk strata.** Cut points were established in the CANARY PASS AS Cohort

| Strata | Combined  Model | Sphingolipid  Panel | PSA  Density | % Positive  Core |
| --- | --- | --- | --- | --- |
| High Risk | >3.2116 | >0.1732 | >0.1293 | >16.67 |
| Intermediate Risk | >2.8988 | >0.1592 | >0.0775 | >8.33 |
| Low-Risk | - | - | - | - |

**Supplementary Table S7. Cox proportional hazard models evaluating association of the sphingolipid panel with GG upgrading in MDACC Set #1.**

|  | **Univariable** | | |
| --- | --- | --- | --- |
| **Variable** | **Hazard Ratio** | **95% CI** | **2-sided P** |
| **Age** | 0.97 | 0.93-1.02 | 0.31 |
| **BMI†** | 0.93 | 0.63-1.38 | 0.72 |
| **PSA Density†** | 1.74 | 1.29-1.94 | 0.0003 |
| **Positive Core Biopsy†** | 1.47 | 1.12-1.94 | 0.006 |
| **Sphingolipid Panel†** | 1.80 | 1.18-2.74 | 0.006 |
| **Sphingolipid Lipid Panel + PSA Density + PCB†** | 3.07 | 2.07-4.54 | <0.0001 |

† per unit standard deviation increase

**Supplementary Table S8. Performance estimates of the sphingolipid panel, PSA density, % positive core biopsy and the model that combines all three at different risk strata for association with GG biopsy upgrade on AS in MDACC Set #1.**

|  |  | **MDACC AS Test Set #1** | | | | | |  |
| --- | --- | --- | --- | --- | --- | --- | --- | --- |
| **Variable** | **Strata** | **# of**  **Events** | **# of**  **Patients** | **Biopsy Upgrade, N (%)** | **No Biopsy Upgrade, N (%)** | **Median time to Upgrade,**  **Months (IQR)** | **P-**  **value†** | **Hazard Ratio**  **(95% CI)** |
| **Combined** | **High-Risk** | **29** | **185** | **22 (34.4)** | **42 (65.6)** | **40.0 (29.9-54.9)** | **<0.0001** | **9.70 (2.89-32.5)** |
|  | **Intermediate-Risk** |  |  | **4 (6.8)** | **55 (93.2)** | **49.6 (20.5-76.7)** |  | **1.48 (0.33-6.60)** |
|  | **Low-Risk** |  |  | **3 (4.8)** | **59 (95.2)** | **50.3 (16.5-60.7)** |  | **reference** |
| Sphingolipid Panel | High-Risk | 29 | 185 | 18 (26.9) | 49 (73.1) | 44.2 (25.6-54.9) | 0.0006 | 6.83 (2.01-23.3) |
|  | Intermediate-Risk |  |  | 8 (14.3) | 48 (85.7) | 37.1 (31.9-65.5) |  | 3.33 (0.88-12.6) |
|  | Low-Risk |  |  | 3 (4.8) | 59 (95.2) | 50.3 (16.5-60.7) |  | reference |
| PSA Density | High-Risk | 29 | 185 | 13 (22) | 46 (78) | 38.1 (28.3-47.1) | 0.142 | 2.24 (0.93-5.42) |
|  | Intermediate-Risk |  |  | 8 (13.1) | 53 (86.9) | 49.6 (37.5-69.0) |  | 0.99 (0.37-2.65) |
|  | Low-Risk |  |  | 8 (12.3) | 57 (87.7) | 44.0 (19.4-64.7) |  | reference |
| % Core Biopsy | High-Risk | 29 | 185 | 9 (26.5) | 25 (73.5) | 46.2 (32.9-66.5) | 0.032 | 2.46 (0.97-6.20) |
|  | Intermediate-Risk |  |  | 11 (16.7) | 55 (83.3) | 52.3 (35.3-60.7) |  | 1.50 (0.62-3.62) |
|  | Low-Risk |  |  | 9 (10.6) | 76 (89.4) | 38.1 (11.9-45.7) |  | reference |

† χ2 test for trend 2-sided p-value

**Supplementary Table S9. Univariable and multivariable Cox proportional hazard models evaluating association of sphingolipid panel with biopsy GG upgrading in MDACC Set #2 (Vykoukal et al. 2020).**

|  | **Univariable** | | | **Multivariable** | | |
| --- | --- | --- | --- | --- | --- | --- |
| **Variable** | **HR** | **95% CI** | **P-value** | **HR** | **95% CI** | **P-value** |
| Age |  |  |  |  |  |  |
| <64 | reference | | | reference | | |
| >=64 | 1.66 | 1.11-2.48 | 0.014 | 2.35 | 1.43-3.86 | 0.0007 |
| BMI† | 1.01 | 0.83-1.23 | 0.91 | 1.09 | 0.86-1.39 | 0.48 |
| Smoking Status |  |  |  |  |  |  |
| No | reference | | | reference | | |
| Yes | 1.13 | 0.75-1.70 | 0.57 | 1.09 | 0.67-1.75 | 0.74 |
| Statin Use |  |  |  |  |  |  |
| No | reference | | | reference | | |
| Yes | 1.26 | 0.85-1.87 | 0.25 | 1.38 | 0.86-2.23 | 0.19 |
| 5-ARI |  |  |  |  |  |  |
| No | reference | | | reference | | |
| Yes | 0.50 | 0.23-1.07 | 0.07 | 0.36 | 0.14-0.91 | 0.03 |
| GGG | 1.09 | 0.68-1.75 | 0.71 | 0.84 | 0.47-1.50 | 0.55 |
| Combined† | 1.28 | 1.06-1.54 | 0.009 | 1.33 | 1.09-1.62 | 0.006 |

† per unit standard deviation increase

Model that combines the Sphingolipid panel + PSA Density + % positive core biopsies

**Supplementary Table S10. Performance estimates of the sphingolipid panel, PSA density, % positive core biopsy and the model that combines all three at different risk strata for association with GG biopsy upgrade on AS in MDACC Set #2 (Vykoukal et al. 2020).**

|  |  | **MDACC Set #2 (Vykoukal et al. 2020)** | | | | | |  |
| --- | --- | --- | --- | --- | --- | --- | --- | --- |
| **Variable** | **Strata** | **# of**  **Events** | **# of**  **Patients** | **Had Biopsy Upgrade, N (%)** | **No Biopsy Upgrade during FU, N (%)** | **Median time to Upgrade,**  **Months (IQR)** | **P-**  **value†** | **Hazard Ratio**  **(95% CI)** |
| **Combined** | **High-Risk** | 73 | 351 | 29 (31.9) | 62 (68.1) | 12.0 (12.0-24.0) | 0.004 | 2.75 (1.54-4.92) |
|  | **Intermediate-Risk** |  |  | 25 (18.5) | 110 (81.5) | 24.0 (12.0-36.0) |  | 1.21 (0.66-2.19) |
|  | **Low-Risk** |  |  | 19 (15.2) | 106 (84.8) | 24.0 (12.0-42.0) |  | reference |
| **Sphingolipid Panel** | **High-Risk** | 98 | 459 | 37 (23.9) | 118 (76.1) | 12.0 (12.0-36.0) | 0.17 | 1.50 (0.92-2.44) |
|  | **Intermediate-Risk** |  |  | 32 (23) | 107 (77) | 18.0 (12.0-36.0) |  | 1.29 (0.78-2.14) |
|  | **Low-Risk** |  |  | 29 (17.6) | 136 (82.4) | 24.0 (12.0-36.0) |  | reference |
| **PSA Density** | **High-Risk** | 98 | 457 | 41 (29.5) | 98 (70.5) | 12.0 (12.0-36.0) | 0.004 | 2.32 (1.43-3.77) |
|  | **Intermediate-Risk** |  |  | 29 (20.6) | 112 (79.4) | 12.0 (12.0-36.0) |  | 1.48 (0.88-2.49) |
|  | **Low-Risk** |  |  | 28 (15.8) | 149 (84.2) | 24.0 (12.0-36.0) |  | reference |
| **% Core Biopsy** | **High-Risk** | 73 | 353 | 17 (34) | 33 (66) | 12.0 (12.0-36.0) | 0.04 | 1.95 (0.45-8.50) |
|  | **Intermediate-Risk** |  |  | 54 (18.2) | 242 (81.8) | 15.0 (12.0-36.0) |  | 0.82 (0.20-3.36) |
|  | **Low-Risk** |  |  | 2 (28.6) | 5 (71.4) | 18.0 (12.0-24.0) |  | reference |

† χ2 test for trend 2-sided p-value

**Supplementary Table S11. Performance estimates of the sphingolipid panel, PSA density, % positive core biopsy and the combined model, stratified by prostate MRI results, in a subset of men in the combined MDACC cohorts (MDACC Set #1 and MDACC Set #2 [Vykoukal et al. 2020]***.* Risk score corresponds to either risk assessment (completed via Likert scoring), PIRADS score or presence of extraprostatic extension. “Low risk” referred to low risk on likert scoring (1-2), PIRADS 1-2, and/or a negative MRI. “High risk” refers to likert scoring (3-5), PIRADS 3-5, and/or suspicion or presence of extraprostatic extension based on the radiologist’s read. Total lesion number refers to total MRI lesions detected by radiologists, irrespective of described risk or PIRADS scoring. Risk strata are the same as defined in **Supplementary Table S6.**

|  |  | **Combined MDACC Cohort - Low risk (PIRADs or Likert Score <= 2 and no EPE noted by radiologist)** | | | | | |  |
| --- | --- | --- | --- | --- | --- | --- | --- | --- |
| **Variable** | **Strata** | **# of** | **# of** | **Biopsy Upgrade, N (%)** | **No Biopsy Upgrade, N (%)** | **Median time to Upgrade,** | **P-** | **Hazard Ratio** |
|  |  | **Events** | **Patients** |  |  | **Months (IQR)** | **value†** | **(95% CI)** |
| **Combined** | High-Risk | 26 | 104 | 8 (44.4) | 10 (55.6) | 12.0 (12.0-24.0) | 0.0003 | 6.33 (2.39-16.80) |
|  | Intermediate-Risk |  |  | 7 (31.8) | 15 (68.2) | 36.0 (18.0-60.0) |  | 2.00 (0.77-5.18) |
|  | Low-Risk |  |  | 11 (17.2) | 53 (82.8) | 36.0 (12.0-60.0) |  | reference |
| Sphingolipid Panel | High-Risk | 32 | 135 | 11 (28.2) | 28 (71.8) | 24.0 (12.0-36.0) | 0.5 | 1.61 (0.70-3.72) |
|  | Intermediate-Risk |  |  | 10 (22.2) | 35 (77.8) | 30.0 (12.0-72.0) |  | 1.09 (0.46-2.56) |
|  | Low-Risk |  |  | 11 (21.6) | 40 (78.4) | 24.0 (12.0-60.0) |  | reference |
| PSA Density | High-Risk | 32 | 125 | 15 (39.5) | 23 (60.5) | 24.0 (12.0-36.0) | 0.005 | 3.08 (1.30-7.31) |
|  | Intermediate-Risk |  |  | 9 (20.5) | 35 (79.5) | 36.0 (18.0-72.0) |  | 1.01 (0.39-2.62) |
|  | Low-Risk |  |  | 8 (18.6) | 35 (81.4) | 15.0 (12.0-36.0) |  | reference |
| % Core Biopsy | High-Risk | 26 | 104 | 13 (30.2) | 30 (69.8) | 24.0 (12.0-42.0) | 0.21 | 1.74 (0.77-3.91) |
|  | Intermediate-Risk |  |  | 2 (15.4) | 11 (84.6) | 18.0 (12.0-24.0) |  | 0.62 (0.14-2.81) |
|  | Low-Risk |  |  | 11 (22.4) | 38 (77.6) | 36.0 (12.0-60.0) |  | reference |
|  |  |  |  |  |  |  |  |  |
|  |  | **Combined MDACC Cohort - High Risk (PIRADs or Likert Score 3+ or EPE noted by radiologist)** | | | | | |  |
| **Variable** | **Strata** | **# of** | **# of** | **Biopsy Upgrade, N (%)** | **No Biopsy Upgrade, N (%)** | **Median time to Upgrade,** | **P-** | **Hazard Ratio** |
|  |  | **Events** | **Patients** |  |  | **Months (IQR)** | **value†** | **(95% CI)** |
| **Combined** | High-Risk | 6 | 18 | 2 (66.7) | 1 (33.3) | 12.0 (12.0-12.0) | 0.09 | 8.82 (0.79-98.1) |
|  | Intermediate-Risk |  |  | 2 (50) | 2 (50) | 42.0 (36.0-48.0) |  | 8.09 (0.64-102.2) |
|  | Low-Risk |  |  | 2 (18.2) | 9 (81.8) | 75.0 (66.0-84.0) |  | reference |
| Sphingolipid Panel | High-Risk | 8 | 24 | 5 (62.5) | 3 (37.5) | 12.0 (12.0-57.0) | 0.21 | 5.40 (0.62-46.80) |
|  | Intermediate-Risk |  |  | 2 (25) | 6 (75) | 48.0 (12.0-84.0) |  | 2.26 (0.20-25.02) |
|  | Low-Risk |  |  | 1 (11.1) | 8 (88.9) | 36.0 (36.0-36.0) |  | reference |
| PSA Density | High-Risk | 8 | 22 | 5 (50) | 5 (50) | 12.0 (12.0-12.0) | 0.26 | 2.69 (0.62-11.69) |
|  | Intermediate-Risk |  |  | 0 (0) | 4 (100) | - |  | - |
|  | Low-Risk |  |  | 3 (37.5) | 5 (62.5) | 66.0 (48.0-84.0) |  | reference |
| % Core Biopsy | High-Risk | 6 | 18 | 4 (57.1) | 3 (42.9) | 51.0 (18.0-84.0) | 0.47 | 1.15 (0.18-7.27) |
|  | Intermediate-Risk |  |  | 0 (0) | 3 (100) | - |  | - |
|  | Low-Risk |  |  | 2 (25) | 6 (75) | 30.0 (12.0-48.0) |  | reference |
|  |  |  |  |  |  |  |  |  |
|  |  | **Combined MDACC Cohort (Number of total MRI Lesions Identified by Radiology [any risk]) = 0)** | | | | | |  |
| **Variable** | **Strata** | **# of** | **# of** | **Biopsy Upgrade, N (%)** | **No Biopsy Upgrade, N (%)** | **Median time to Upgrade,** | **P-** | **Hazard Ratio** |
|  |  | **Events** | **Patients** |  |  | **Months (IQR)** | **value†** | **(95% CI)** |
| **Combined** | High-Risk | 14 | 46 | 5 (71.4) | 2 (28.6) | 12.0 (12.0-36.0) | 0.00018 | 12.13 (3.08-47.78) |
|  | Intermediate-Risk |  |  | 3 (37.5) | 5 (62.5) | 36.0 (12.0-72.0) |  | 3.48 (0.82-14.79) |
|  | Low-Risk |  |  | 6 (19.4) | 25 (80.6) | 30.0 (21.0-75.0) |  | reference |
| Sphingolipid Panel | High-Risk | 15 | 56 | 6 (33.3) | 12 (66.7) | 12.0 (12.0-39.0) | 0.49 | 1.90 (0.58-6.24) |
|  | Intermediate-Risk |  |  | 4 (25) | 12 (75) | 42.0 (18.0-66.0) |  | 1.02 (0.27-3.79) |
|  | Low-Risk |  |  | 5 (22.7) | 17 (77.3) | 24.0 (18.0-54.0) |  | reference |
| PSA Density | High-Risk | 15 | 38 | 7 (70) | 3 (30) | 12.0 (12.0-48.0) | 0.0023 | 7.00 (1.80-27.11) |
|  | Intermediate-Risk |  |  | 5 (25) | 15 (75) | 36.0 (24.0-78.0) |  | 1.66 (0.40-6.95) |
|  | Low-Risk |  |  | 3 (13) | 20 (87) | 12.0 (12.0-36.0) |  | reference |
| % Core Biopsy | High-Risk | 14 | 46 | 5 (35.7) | 9 (64.3) | 36.0 (18.0-78.0) | 0.79 | 1.31 (0.41-4.17) |
|  | Intermediate-Risk |  |  | 2 (22.2) | 7 (77.8) | 18.0 (12.0-24.0) |  | 0.78 (0.16-3.76) |
|  | Low-Risk |  |  | 7 (30.4) | 16 (69.6) | 24.0 (12.0-48.0) |  | reference |
|  |  |  |  |  |  |  |  |  |
|  |  | **Combined MDACC Cohort (Number of total MRI Lesions Identified by Radiology [any risk])=1+** | | | | | |  |
| **Variable** | **Strata** | **# of** | **# of** | **Biopsy Upgrade, N (%)** | **No Biopsy Upgrade, N (%)** | **Median time to Upgrade,** | **P-** | **Hazard Ratio** |
|  |  | **Events** | **Patients** |  |  | **Months (IQR)** | **value†** | **(95% CI)** |
| **Combined** | High-Risk | 12 | 58 | 3 (27.3) | 8 (72.7) | 12.0 (12.0-24.0) | 0.23 | 3.53 (0.79-15.84) |
|  | Intermediate-Risk |  |  | 4 (28.6) | 10 (71.4) | 36.0 (22.5-54.0) |  | 1.46 (0.39-5.45) |
|  | Low-Risk |  |  | 5 (15.2) | 28 (84.8) | 36.0 (12.0-54.0) |  | reference |
| Sphingolipid Panel | High-Risk | 17 | 79 | 5 (23.8) | 16 (76.2) | 36.0 (18.0-48.0) | 0.89 | 1.36 (0.41-4.46) |
|  | Intermediate-Risk |  |  | 6 (20.7) | 23 (79.3) | 21.0 (12.0-36.0) |  | 1.21 (0.39-3.77) |
|  | Low-Risk |  |  | 6 (20.7) | 23 (79.3) | 36.0 (12.0-63.0) |  | reference |
| PSA Density | High-Risk | 17 | 72 | 8 (28.6) | 20 (71.4) | 30.0 (24.0-36.0) | 0.29 | 1.65 (0.53-5.10) |
|  | Intermediate-Risk |  |  | 4 (16.7) | 20 (83.3) | 36.0 (12.0-69.0) |  | 0.65 (0.17-2.42) |
|  | Low-Risk |  |  | 5 (25) | 15 (75) | 18.0 (12.0-42.0) |  | reference |
| % Core Biopsy | High-Risk | 12 | 58 | 8 (28.6) | 20 (71.4) | 21.0 (12.0-36.0) | 0.11 | 2.56 (0.76-8.65) |
|  | Intermediate-Risk |  |  | 0 (0) | 4 (100) | - |  | - |
|  | Low-Risk |  |  | 4 (15.4) | 22 (84.6) | 48.0 (18.0-60.0) |  | reference |

† χ2 test for trend 2-sided p-value

**Supplementary Table S12. Power calculation for training and two validation cohorts.**

|  |  | **Hazard ratio** | **# of events in elevated risk group** | **# of events in low-risk group** | **Total # of events** | **Power** | **Type one error** | **Method** |
| --- | --- | --- | --- | --- | --- | --- | --- | --- |
| **Cohort** | **MDACC Set #1** | 9.7 | 22 | 3 | 25 | 0.99 | 0.05 | **Power analysis of Log-rank test (Schoenfeld's formula) (Schoenfeld, D. (1981))** |
|  |  | 1.48 | 4 | 3 | 7 | 0.07 | 0.05 |  |
|  | **MDACC Set #2** | 2.75 | 29 | 19 | 48 | 0.94 | 0.05 |  |
|  |  | 1.21 | 25 | 19 | 44 | 0.09 | 0.05 |  |
|  | **PASS** | 3.17 | 42 | 19 | 61 | 0.99 | 0.05 |  |
|  |  | 2.05 | 37 | 19 | 56 | 0.77 | 0.05 |  |

Each row represents power calculation for detectable change in the respective second and third score tertiles, with the first (lowest) tertile being the referrant group.

**Supplementary Table S13. Patient characteristics in all cohorts stratified by progression status**

| **Variable** | **Canary PASS** | | **MDACC Set #1** | | **MDACC Set #2** | |
| --- | --- | --- | --- | --- | --- | --- |
|  | **Progress** | **No Progress** | **Progress** | **No Progress** | **Progress** | **No Progress** |
| **N** | 98 | 446 | 33 | 215 | 98 | 361 |
| **Age, yrs (median, IQR)** | 65 (60-70) | 64 (59-69) | 62 (57-65) | 63 (59-68) | 65 (60-70) | 63 (57-69) |
| **PSA Density (median, IQR)** | 0.11 (0.069-0.15) | 0.10 (0.06-0.14) | 0.12 (0.07-0.24) | 0.10 (0.06-0.15) | 0.11 (0.07-0.16) | 0.09 (0.05-0.13) |
| **Highest Gleason Grade Group, diagnostic or confirmatory biopsy (N, %)** |  |  |  |  |  |  |
| **1** | 85 (86.7) | 382 (85.7) | 29 (87.9 | 185 (86.0) | 83 (84.7) | 293 (81.2) |
| **2** | 12 (12.2) | 62 (13.9) | 3 (9.1) | 24 (11.2) | 12 (12.2) | 64 (17.7) |
| **3** | 1 (<1) | 2 (<1) | 1 (3.0) | 6 (2.8) | 3 (3.1) | 4 (1.1) |
| **Percentage of positive cores at diagnosis (median, IQR)** | 8.3 (8.3-16.7) | 8.3 (8.3-16.7) | 16.7 (8.3-25.0) | 9.1 (8.3-16.7) | 13.3 (8.3-16.7) | 8.3 (8.3-16.7) |
| **BMI (median, IQR)** | 27.8 (25.7- 30.7) | 27.8 (25.5-30.9) | 28.4 (25.9-30.9) | 29.1 (26.5-32.7) | 29.0 (26.1-31.3) | 28.5 (26.0-31.6) |
| **Race** |  |  |  |  |  |  |
| **White** | 88 (89.8) | 389 (87.2) | 28 (84.8) | 160 (74.4) | 37 (80.4) | 295 (81.7) |
| **Black** | 4 (4.1) | 34 (7.6) | 1 (<1) | 17 (7.9) | 3 (6.5) | 28 (7.8) |
| **Other/Unknown** | 6 (6.1) | 23 (5.2) | 3 or 4 (12.1) | 38 (17.7) | 6 (13.0) | 38 (10.5) |
| **Statin (N, %)** |  |  |  |  |  |  |
| **Yes** | 39 (39.8) | 199 (44.6) |  |  | 52 (53.1) | 165 (45.7) |
| **No** | 59 (60.2) | 247 (55.4) |  |  | 46 (46.9) | 196 (54.3) |
| **Diabetes (N,%)*** |  |  |  |  |  |  |
| **Yes** | 7 (7.1) | 41 (9.2) |  |  |  |  |
| **No** | 91 (92.9) | 405 (90.8) |  |  |  |  |
| **Follow-up, yrs (median, IQR)** | 2.6 (1.6-3.6) | 2.2 (1.1-4.5) | 3.5 (2.3-4.4) | 3.4 (1.0-5.1) | 1.5 (1.0-3.0) | 3.0 (2.0-5.0) |

**Figure S1. Schematic workflow of the model development.**

**Specimen Set**

782 men diagnosed with localized prostate cancer who opted for active surveillance

**Training Set (PASS)**

544 men with localized prostate cancer

# of progressed cases: 98

**MDACC Set #1**

238 men with localized prostate cancer

# of progressed cases: 33

**8 Models Evaluated**

1- Neural Network (from H2O Package)

2- akritas (Conditional Non-Parametric Survival Estimator)

3- coxtime (Cox-Time Survival Neural Network)

4- Coxph

5- DeepHit Survival Neural Network

6- Deepsurv DeepSurv Survival Neural Network

7- Logistic-Hazard Survival Neural Network

8 - PC-Hazard Survival Neural Network

**Stability check (Predictability, stability and reproducibility)**

Combined Lipid Panel+PSA Density+ Prostate Core Biopsy Positivity Model **(Cox Regression)**

**A deep learning model (DLM) with 3 hidden layers and 6 nodes in each layer selected for modeling the 20-marker metabolite panel**

**MDACC Set #2***

459 men with localized prostate cancer,

# of progressed cases: 98

*MDACC Set #2 consisted of patients included in initial plasma sphingolipid evaluation completed by our group (Vykoukal et al. Nat Comms 2020). This set is included in the current work as verification of sphingolipid panel performance but was not used for primary validation.

**Figure S2. Association between sphingolipid panel scores and BMI (A) and statin use (B) in the PASS Cohort. A)** Scatter plot illustrating relationship between sphingolipid panel scores and BMI among AS patients that had GG upgrading (red nodes) and those that did not (blue nodes). **B)** Violin plots illustrating the distribution of sphingolipid panel scores among AS patients who were on statin use versus those that did not use statins.

**
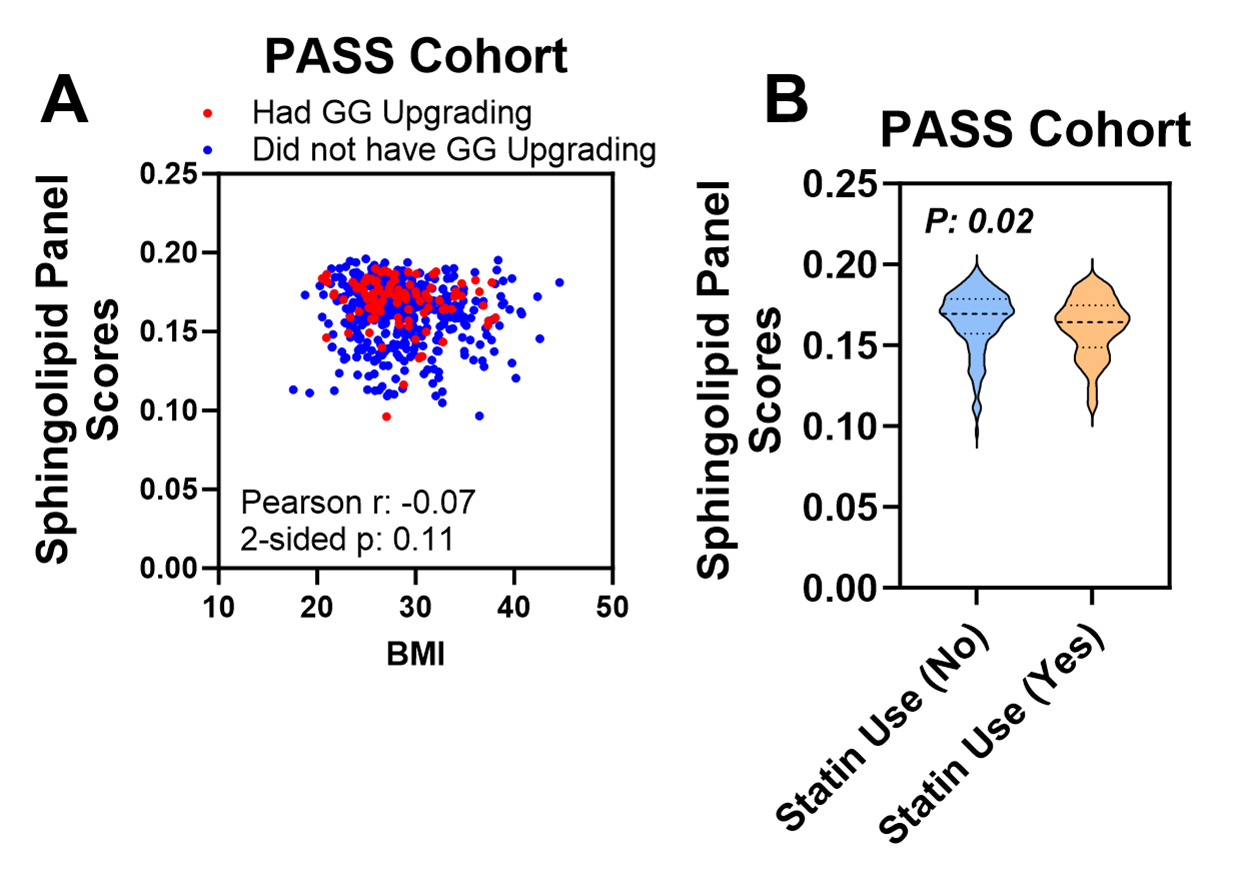
**

**Figure S3. Correlation heatmap showing association between sphingolipid panel score, PSA density, and positive core biopsy rates.**

**
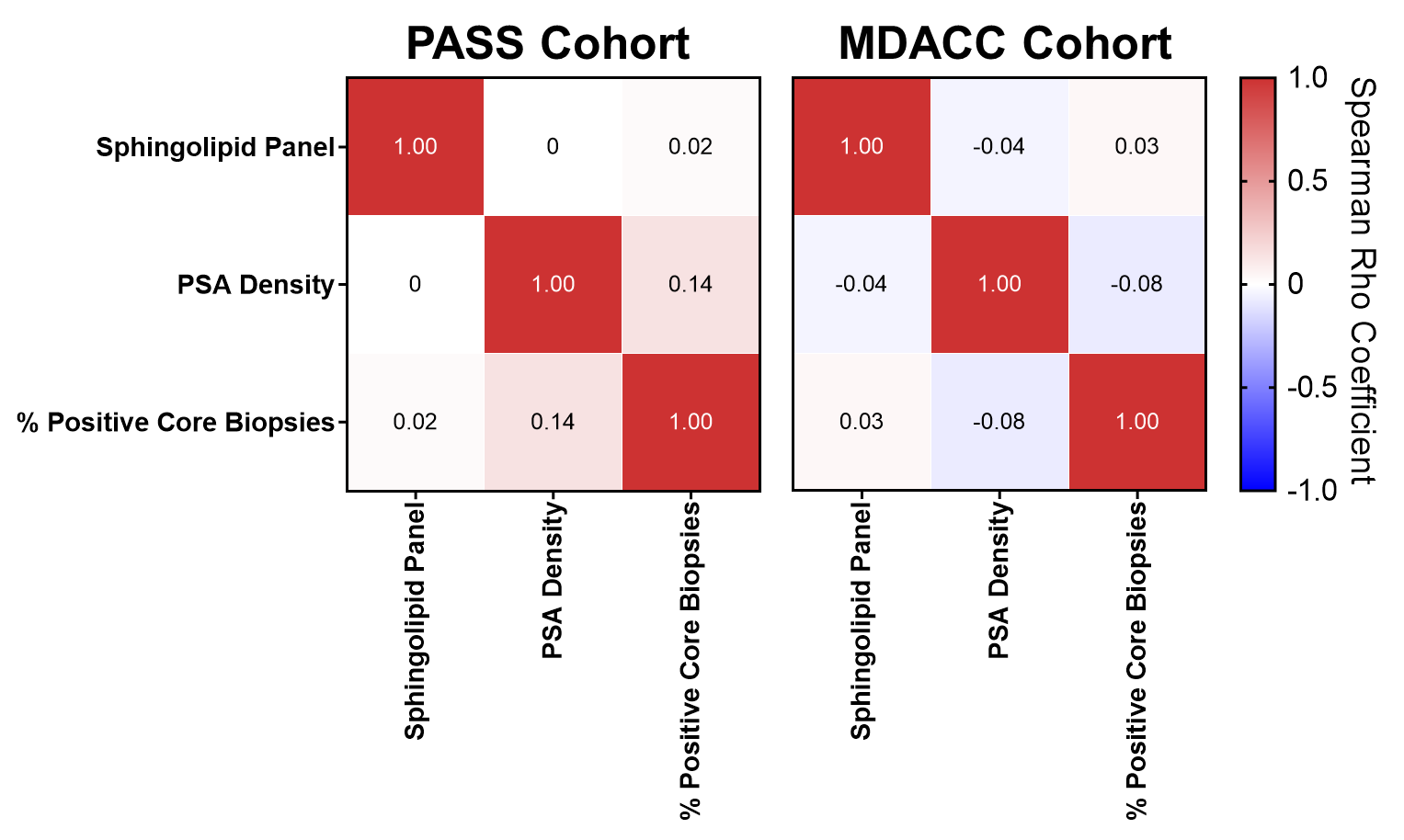
**

**Figure S4. AUC curves demonstrating Predictive performance estimates of the combined model (sphingolipid panel, PSAD and % core positive) for GG biopsy upgrade in the PASS Cohort, MDACC Set #1, MDACC Set #2 and the combined MDACC (Set #1 and Set #2) Cohorts**
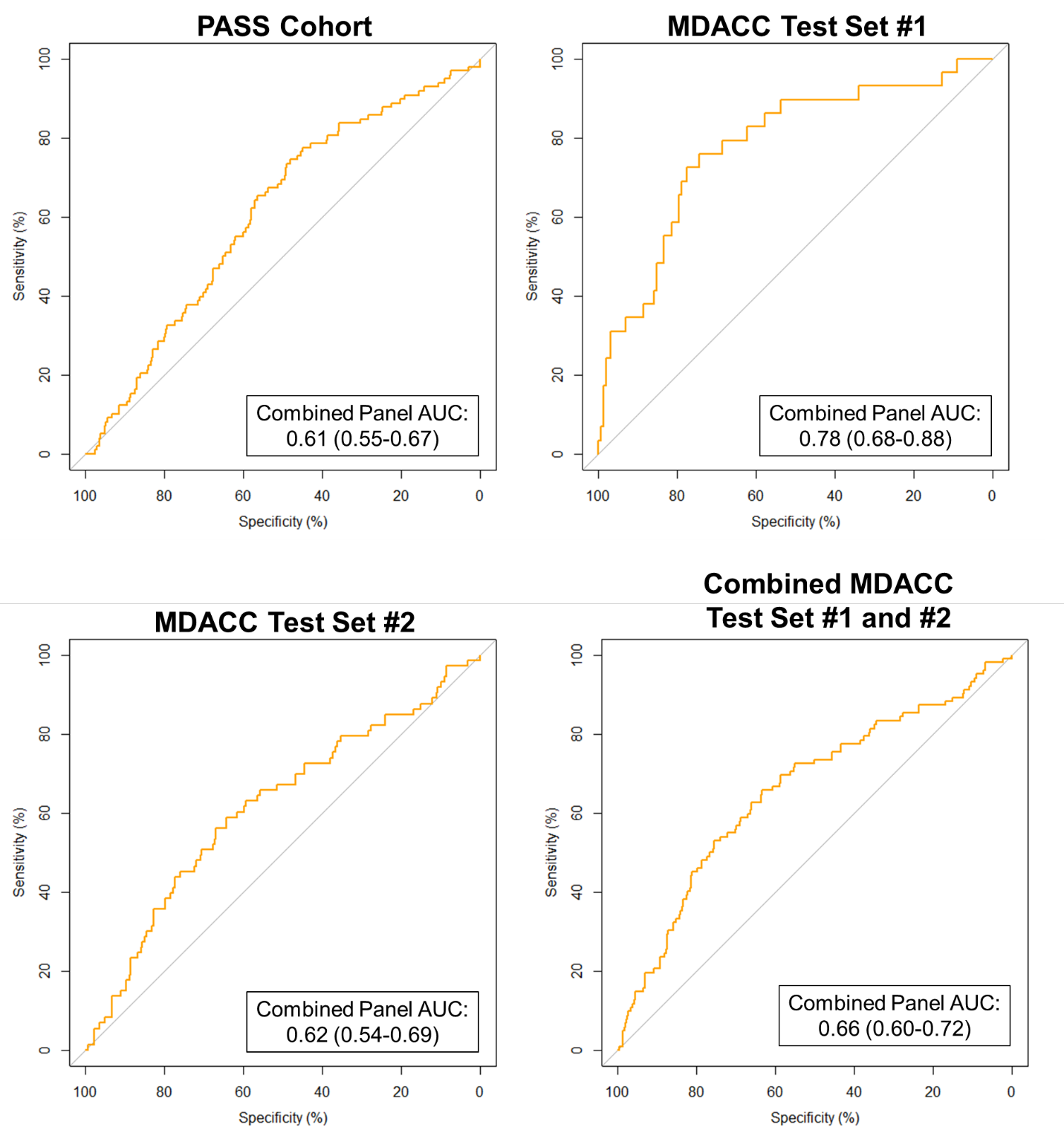


**Figure S5. Cumulative incidence curves for biopsy upgrade based on combined model at high-, intermediate-, and low-risk strata in MDACC Set #1.** Risk tables, including censoring events, are provided beneath. Censoring was attributed to GG upgrading, prostate cancer treatment, voluntary withdrawal from AS, or loss to follow-up following biopsy.

**
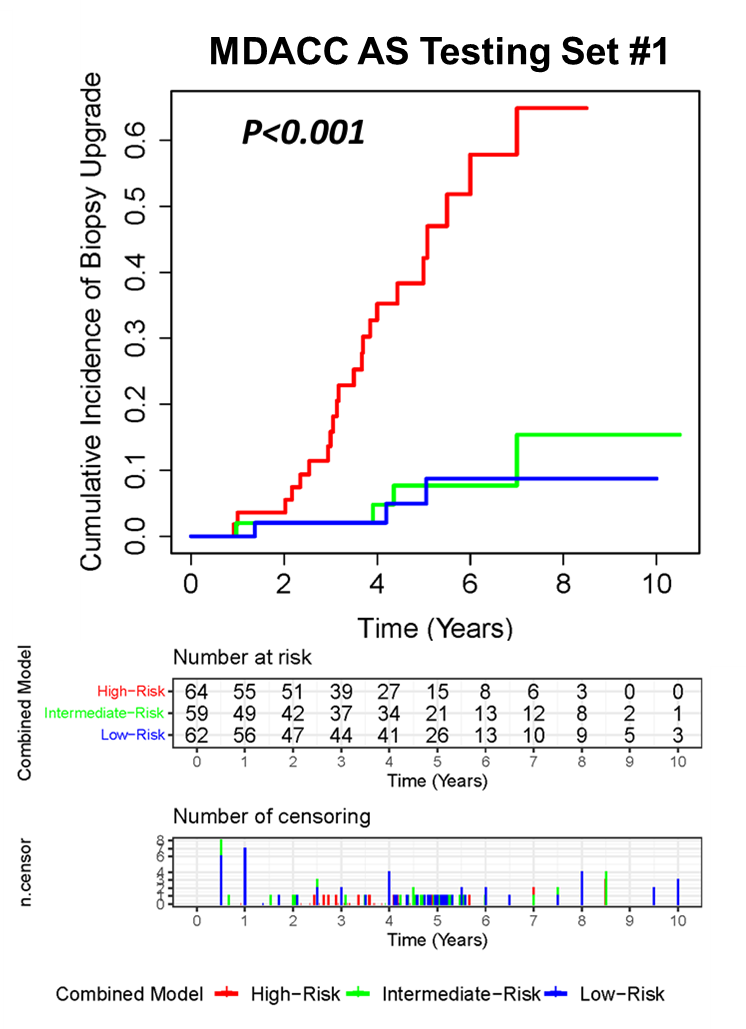
**

**Figure S6. Cumulative incidence curves for biopsy upgrade based on combined model at high-, intermediate-, and low-risk strata in the MDACC Set #2 [Vykoukal et al. 2020]).** Risk tables, including censoring events, are provided beneath. Censoring was attributed to GG upgrading, prostate cancer treatment, voluntary withdrawal from AS, or loss to follow-up following biopsy.

**
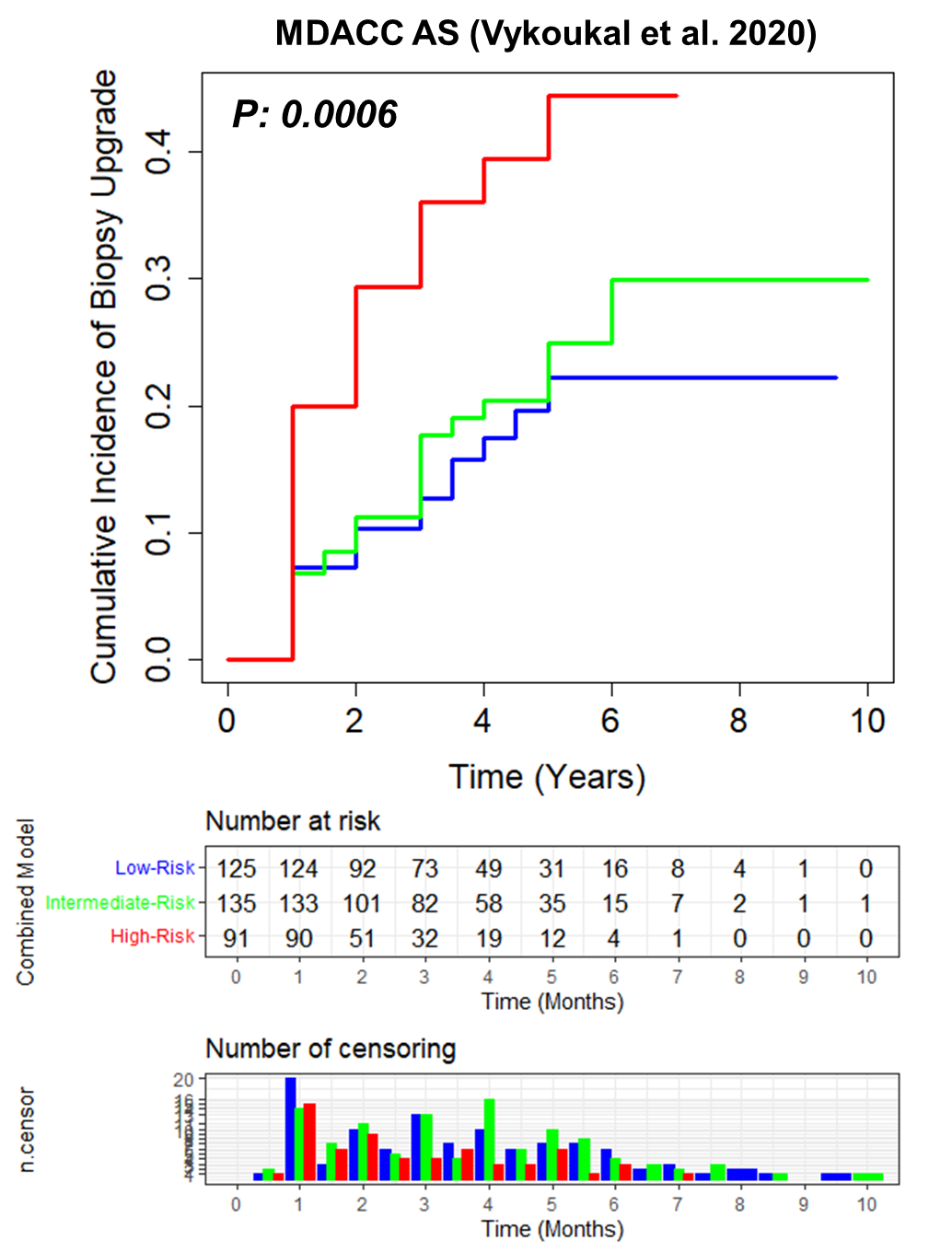
**

**Figure S7. Cumulative incidence curves for biopsy upgrade based** **PIRADS score 0-2 versus PIRADS score 3+ in a subset of men in the combined MDACC cohorts (Testing Set #1 and Testing Set #2 [Vykoukal et al. 2020]***.* Risk tables, including censoring events, are provided beneath. Censoring was attributed to GG upgrading, prostate cancer treatment, voluntary withdrawal from AS, or loss to follow-up following biopsy.

**
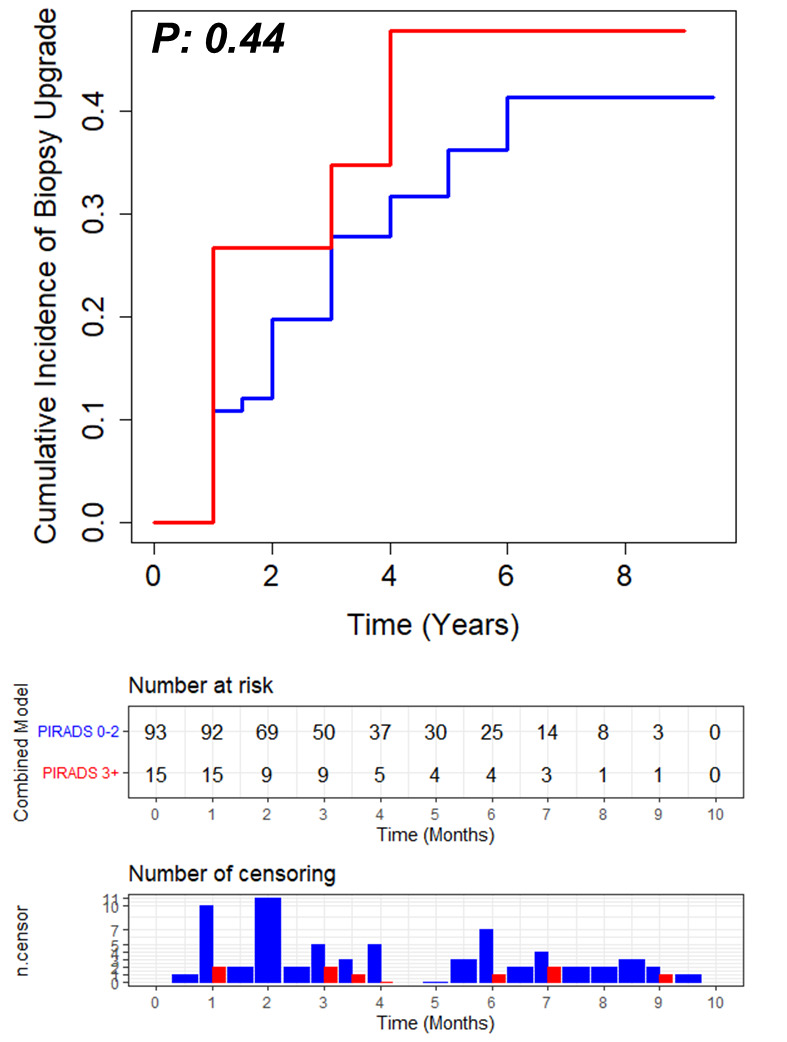
**
